# Supplementary material for: Observation of nonlinear thermoelectric effect in MoGe/Y3Fe5O12
Source: Nat Commun. 2024 Aug 26;15:6912. doi: 10.1038/s41467-024-50115-4 (PMC11347653; doi:10.1038/s41467-024-50115-4)
Supplement: Supplementary file 1 — Supplementary Information [file 41467_2024_50115_MOESM1_ESM.pdf]

Supplementary Information for

Observation of nonlinear thermoelectric effect in MoGe/$Y_3Fe_5O_{12}$

Hiroki Arisawa,[1,2,3] Yuto Fujimoto,[1] Takashi Kikkawa,[1] and Eiji Saitoh[1,2,4,5,*]

[1] *Department of Applied Physics, The University of Tokyo, Tokyo 113-8656, Japan.*

[2] *RIKEN Center for Emergent Matter Science, Wako 351-0198, Japan.*

[3] *Institute for Materials Research, Tohoku University, Sendai 980-8577, Japan.*

[4] *Institute for AI and Beyond, The University of Tokyo, Tokyo 113-8656, Japan.*

[5] *WPI, Advanced Institute for Materials Research, Tohoku University, Sendai 980-8577, Japan.*

Table of Contents

Supplementary Note 1 | Measurement principle to detect nonlinear vortex Nernst effect.

Supplementary Note 2 | Theoretical model for nonlinear vortex Nernst effect.

2.1 Free energy for a vortex string

2.2 Vortex nucleation at surfaces

2.3 Nonlinear vortex Nernst effect

Supplementary Note 3 | Comparison of thermoelectric response between MoGe/YIG and MoGe/SiO_2 samples.

Supplementary Note 4 | Evaluation of uniform heating in MoGe/YIG sample.

Supplementary Note 5 | Influence of uniform heating in permalloy sample.

Supplementary Note 6 | Evaluation of temperature inhomogeneity in MoGe/YIG sample.

Supplementary Note 7 | Out-of-plane and in-plane temperature gradients in MoGe/YIG sample.

Supplementary Note 8 | Out-of-plane temperature gradient in permalloy sample.

Supplementary Note 9 | Frequency dependence of thermoelectric voltage in MoGe/YIG sample.

Supplementary References

Supplementary Note 1 | Measurement principle to detect nonlinear vortex Nernst effect.

The measurement of nonlinear thermoelectric voltages was performed based on a lock-in technique. When a temperature difference ΔT is applied to a material, the generated thermoelectric voltage V can be expanded as

$$V = a_{1\mathrm{st}}\Delta T + a_{2\mathrm{nd}}(\Delta T)^2 + \cdots, \quad \text{(S1)}$$

where $a_{n-\mathrm{th}}$ $(n = 1, 2, \cdots)$ is the n-th order thermoelectric coefficient. When the input temperature difference oscillates at the frequency $\omega/2\pi$, the n-th harmonic signal detected with a lock-in amplifier is given by

$$V_{n\omega}(t) = \frac{1}{t_0}\int_{t-t_0}^{t} \sin(n\omega t' + \theta_0)V(t')dt', \quad \text{(S2)}$$

where t_0 is a sufficiently large averaging time and θ_0 is a set phase for the amplifier.

In conventional linear thermoelectric measurement, a temperature difference is generated by the Joule heating, $\Delta T \propto \sin^2 \omega t$, due to the a.c. current $\propto \sin \omega t$ applied to a heater attached to an end of the sample, and a 2ω lock-in voltage is measured. Similarly, one might think that a nonlinear thermoelectric effect $\propto (\Delta T)^2$ can be measured via the 4ω lock-in measurement. However, the 4ω lock-in signal may include not only the second harmonic response but also the third harmonic one which may appear even without inversion symmetry breaking. To address this issue, the order of the lock-in detection should be lowered, which is achieved if the input temperature difference oscillates in a first-harmonic manner, $\Delta T \propto \sin \omega t$.

We have developed a method to create $\Delta T \propto \sin \omega t$ and detect the second-order nonlinear thermoelectric voltage exclusively. Two heaters are attached to the top and bottom surfaces of the sample and the other sides of the heaters are connected to a heat bath (Supplementary Fig. 1). Then, a.c. and d.c. currents are applied to each heater: $I_{\mathrm{H1}} \sin(\omega t + \phi) + I_{\mathrm{DC1}}$ ($I_{\mathrm{H2}} \sin\omega t + I_{\mathrm{DC2}}$) to the Heater-1 (Heater-2). Due to the Joule heating, ΔT is generated:

$$\begin{aligned} \Delta T &= R_1[I_{\mathrm{H1}} \sin(\omega t + \phi) + I_{\mathrm{DC1}}]^2 - R_2(I_{\mathrm{H2}}\sin\omega t + I_{\mathrm{DC2}})^2 \\ &= \tilde{I}_{\mathrm{DC1}}^2 - \tilde{I}_{\mathrm{DC2}}^2 + 2\tilde{I}_{\mathrm{DC1}}\tilde{I}_{\mathrm{H1}} \sin(\omega t + \phi) - 2\tilde{I}_{\mathrm{DC2}}\tilde{I}_{\mathrm{H2}} \sin \omega t + \tilde{I}_{\mathrm{H1}}^2\sin^2(\omega t + \phi) - \tilde{I}_{\mathrm{H2}}^2\sin^2\omega t, \end{aligned} \quad \text{(S3)}$$

where R_1 (R_2) represents the prefactor relating the Joule heating of the Heater-1 (Heater-2) to ΔT, and $\tilde{I}_{\mathrm{H1,DC1}}$ ($\tilde{I}_{\mathrm{H2,DC2}}$) is defined as $\tilde{I}^2{}_{\mathrm{H1,DC1}} = R_1 I_{\mathrm{H1,DC1}}^2$ ($\tilde{I}^2{}_{\mathrm{H2,DC2}} = R_2 I_{\mathrm{H2,DC2}}^2$). When the power of each heater is the same ($\tilde{I}_{\mathrm{DC1}} = \tilde{I}_{\mathrm{DC2}} = \tilde{I}_{\mathrm{DC}}$ and $\tilde{I}_{\mathrm{H1}} = \tilde{I}_{\mathrm{H2}} = \tilde{I}_{\mathrm{H}}$), Eq. (S3) becomes

$$\Delta T = 2\tilde{I}_{\mathrm{DC}}\tilde{I}_{\mathrm{H}}[\sin(\omega t + \phi) - \sin \omega t] + \tilde{I}_{\mathrm{H}}^2[\sin^2(\omega t + \phi) - \sin^2 \omega t]. \quad \text{(S4)}$$

At $\phi = 0$, ΔT vanishes all the time. In contrast, at $\phi = \pi$, the first and second terms are enhanced while the third and fourth terms cancel out each other, and $\Delta T \propto \sin\omega t$ is achieved.

We give a procedure to optimize ΔT [from Eq. (S3) to Eq. (S4)] and apply it to the present system: a type-II superconductor MoGe and a ferrimagnetic insulator $Y_3Fe_5O_{12}$ (YIG) bilayer structure on a $Gd_3Ga_5O_{12}$ (GGG) substrate (see Supplementary Fig. 1). The essence is to find a value of $(\tilde{I}_{\mathrm{DC1}}, \tilde{I}_{\mathrm{DC2}}, \tilde{I}_{\mathrm{H1}}, \tilde{I}_{\mathrm{H2}})$ where both the 1ω and 2ω lock-in voltages ($V_{1\omega}$ and $V_{2\omega}$, respectively) due to the "linear" vortex Nernst effect disappear, which corresponds to the situation of $\Delta T = 0$ at an arbitrary time. First, we set the relative phase of the heater currents to be zero ($\phi = 0$). A possible phase shift ϕ' between the input a.c. current and the resultant temperature difference in the sample, if present, should be calibrated here, which can be done by measuring the ϕ dependence of the 2ω lock-in voltage $V_{2\omega}$ due to the linear vortex Nernst effect ($\propto \Delta T$) arising from the fifth and sixth terms in Eq. (S3), followed by a $\sin^2(\phi + \phi')$ fit to the result. Under the situation at $\phi = 0$, we minimize the experimentally obtained $V_{2\omega}$ [$\propto \Delta T \propto (\tilde{I}_{\mathrm{H1}}^2 - \tilde{I}_{\mathrm{H2}}^2)\sin^2\omega t$ in Eq. (S3)] by tuning the values of I_{H1} and I_{H2} to find the condition $\tilde{I}_{\mathrm{H1}} = \tilde{I}_{\mathrm{H2}} = \tilde{I}_{\mathrm{H}}$. After the calibration, ΔT is given by

$$\Delta T = \tilde{I}_{\mathrm{DC1}}^2 - \tilde{I}_{\mathrm{DC2}}^2 + 2\tilde{I}_{\mathrm{H}}(\tilde{I}_{\mathrm{DC1}} - \tilde{I}_{\mathrm{DC2}}) \sin\omega t. \quad \text{(S5)}$$

Next, we minimize the 1ω lock-in voltage $V_{1\omega} \propto \Delta T \propto \tilde{I}_{\mathrm{H}}(\tilde{I}_{\mathrm{DC1}} - \tilde{I}_{\mathrm{DC2}}) \sin\omega t$ [third and fourth terms in Eq. (S5)] by tuning the values of I_{DC1} and I_{DC2}, and find the condition $\tilde{I}_{\mathrm{DC1}} = \tilde{I}_{\mathrm{DC2}} = \tilde{I}_{\mathrm{DC}}$. Under these optimized conditions ($\tilde{I}_{\mathrm{H1}} = \tilde{I}_{\mathrm{H2}} = \tilde{I}_{\mathrm{H}}$ and $\tilde{I}_{\mathrm{DC1}} = \tilde{I}_{\mathrm{DC2}} = \tilde{I}_{\mathrm{DC}}$), the temperature difference across the sample is kept zero ($\Delta T = 0$) for $\phi = 0$, and at an arbitrary value of ϕ, the temperature difference given by Eq. (S4) is established. In particular, when $\phi = \pi$, the temperature difference oscillating at 1ω is produced across the sample without 2ω components: $\Delta T = -4\tilde{I}_{\mathrm{DC}}\tilde{I}_{\mathrm{H}} \sin\omega t$, realizing the pure detection of the nonlinear vortex Nernst effect $\propto (\Delta T)^2$ via the measurement of the 2ω lock-in voltage (Fig. 2e in the main text). Furthermore, we found that the 2ω lock-in voltage $V_{2\omega}^{\mathrm{2nd}}$ due to the second-order nonlinear thermoelectric effect $\propto (\Delta T)^2 \propto [\sin(\omega t + \phi) - \sin\omega t]^2$ obeys a characteristic ϕ dependence [$V_{2\omega}^{\mathrm{2nd}} \propto a_{\mathrm{2nd}}\cos\phi\sin^2(\phi/2)$], which is distinct from the ϕ dependence of the 2ω lock-in voltage $V_{2\omega}^{\mathrm{1st}}$ due to the "linear" thermoelectric effect [$V_{2\omega}^{\mathrm{1st}} \propto a_{\mathrm{1st}} \sin^2\phi$] arising from $\Delta T \propto [\sin^2(\omega t + \phi) - \sin^2\omega t]$. The characteristic allows clear separation between the nonlinear and linear thermoelectric voltages in terms of the ϕ dependence of $V_{2\omega}$.

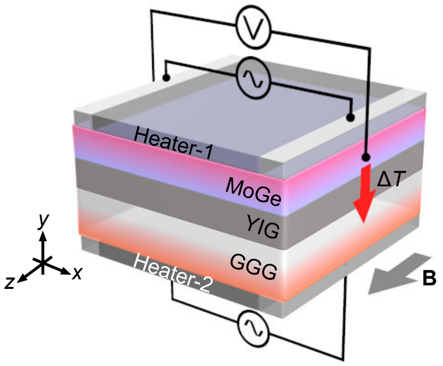

Supplementary Fig. 1 Schematic illustration of measurement setup. A MoGe/YIG/GGG sample was sandwiched by two heaters (Heater-1 and Heater-2). The a.c. and d.c. currents $I_{\mathrm{H1}}\sin(\omega t+\phi)+I_{\mathrm{DC1}}$ ($I_{\mathrm{H2}}\sin\omega t+I_{\mathrm{DC2}}$) were applied to the Heater-1 (Heater-2). A temperature difference ΔT was generated along the y axis by the Joule heating from the Heater-1 and the Heater-2. The voltage between the ends of the MoGe film in the x direction was measured with a lock-in amplifier under the external magnetic field **B** along the z axis.

Supplementary Note 2 | Theoretical model for nonlinear vortex Nernst effect.

2.1 Free energy for a vortex string

We consider a mobile vortex system in a type-II superconductor on a magnetic insulator, in which vortex-string dynamics and its nucleation play a role (see Supplementary Fig. 2).

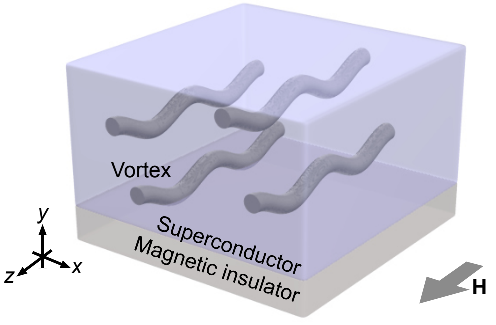

Supplementary Fig. 2 Vortex system in type-II superconductor on magnetic insulator. Vortex strings are aligned along the magnetic field **H** in the z direction.

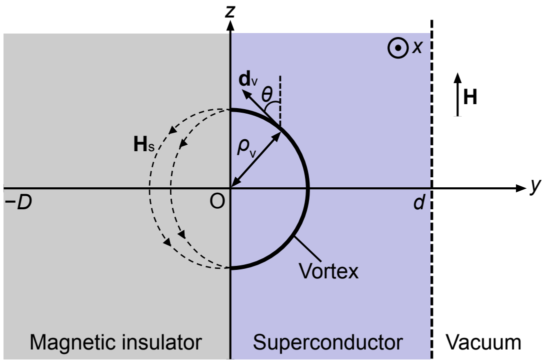

Supplementary Fig. 3 Calculation model of vortex nucleation at superconductor surface. A magnetic insulator, a superconductor, and vacuum are occupied in $-D \leq y \leq 0$, $0 \leq y \leq d$, and $d \leq y$, respectively. The magnetic field **H** is applied in the z direction. $\mathbf{H}_\mathrm{s}$, $\mathbf{d}_\mathrm{v}$, θ, and ρ_v denote a stray magnetic field from the vortex string endpoints, a unit tangent vector along the vortex string, the relative angle between $\mathbf{d}_\mathrm{v}$ and the z axis, and the radius of a semicircle vortex, respectively.

For simplicity, we assume that a vortex string is nucleated in a semicircle form at the superconductor surface (Supplementary Fig. 3). The free energy of the vortex in this case can be expressed as [S1,S2]

$$G \sim \frac{\Phi_0 H_\mathrm{v} \lambda}{4\pi}\left(\frac{\rho_\mathrm{v}}{\lambda}\right)\left[\left(\frac{\pi}{2}-\frac{2H}{H_\mathrm{v}}\right)+\frac{2H}{H_\mathrm{v}}\int_0^{\frac{\pi}{2}} d\theta \cos\theta \exp\left(-\frac{\rho_\mathrm{v}}{\lambda}\cos\theta\right)\right], \tag{S6}$$

where Φ_0 is the quantum flux, λ is the magnetic-field penetration depth, $H_\mathrm{v} \sim \Phi_0/\lambda^2$ is the magnetic field at the vortex core, ρ_v is the radius of a semicircle vortex, H is the external magnetic field, and θ is the relative angle between a unit tangent vector $\mathbf{d}_\mathrm{v}$ along the vortex string and the z axis (see Supplementary Fig. 3).

Next, we consider the influence of the magnetic insulator on the free energy. The magnetization $\mathbf{M}$ of the magnetic insulator interacts with the stray magnetic field $\mathbf{H}_\mathrm{s}$ from the vortex string at the interface. This stray magnetic field is assumed to satisfy the Maxwell equations with the boundary condition

$$\nabla \cdot \mathbf{H}_\mathrm{s} = 0, \tag{S7}$$

$$\nabla \times \mathbf{H}_\mathrm{s} = 0, \tag{S8}$$

$$\mathbf{H}_\mathrm{s} = \mathbf{H}_\mathrm{v} \quad (y = 0). \tag{S9}$$

In the limit of $r = \sqrt{x^2 + y^2 + z^2} \gg \rho_\mathrm{v}$, the solution of $\mathbf{H}_\mathrm{s}$ is given by the dipole magnetic field generated from the endpoints of the semicircle vortex string at the interface [S2]:

$$\mathbf{H}_\mathrm{s} = -\nabla U_\mathrm{d}, \quad U_\mathrm{d} = 2\rho_\mathrm{v}\Phi_0 \frac{z}{r^3}. \tag{S10}$$

The interaction between the stray magnetic field $\mathbf{H}_\mathrm{s} = ([H_\mathrm{s}]_x, [H_\mathrm{s}]_y, [H_\mathrm{s}]_z)$ and the magnetization $\mathbf{M}$ of the magnetic insulator contributes to the vortex free energy given by

$$F_\mathrm{mag} = -\int d^3r \mathbf{H}_\mathrm{s} \cdot \mathbf{M}. \tag{S11}$$

Under an external magnetic field $\mathbf{H}$ in the z direction ($|\mathbf{H}| \gg |\mathbf{H}_\mathrm{s}|$), the magnetization is decomposed into

$$\mathbf{M} = \mathbf{M}_\parallel + \delta\mathbf{M}_\perp, \tag{S12}$$

where $\mathbf{M}_\parallel = \chi_\parallel \mathbf{H}$ is the longitudinal magnetization parallel to the external magnetic field and $\delta\mathbf{M}_\perp = \chi_\perp \mathbf{H}_{\mathrm{s}\perp}$ is the transverse magnetization due to the stray field $\mathbf{H}_{\mathrm{s}\perp} = ([H_\mathrm{s}]_x, [H_\mathrm{s}]_y, 0)$. $\chi_\parallel$ ($\chi_\perp$) represents the longitudinal (transverse) susceptibility in the magnetic insulator. By substituting Eq. (S12) into Eq. (S11), we obtain

$$F_\mathrm{mag} = -M_\parallel \int d^3r\, [H_\mathrm{s}]_z - \int d^3r\, \mathbf{H}_{\mathrm{s}\perp} \cdot \delta\mathbf{M}_\perp \tag{S13}$$

$$= -M_\parallel \int d^3r\, [H_\mathrm{s}]_z - \chi_\perp \int d^3r\, \left([H_\mathrm{s}]_x{}^2 + [H_\mathrm{s}]_y{}^2\right) = F_\parallel + F_\perp. \tag{S14}$$

Substitution of Eq. (S10) into Eq. (S14) leads to the longitudinal term as [S1]

$$F_\parallel \sim M_\parallel \Phi_0 \rho_\mathrm{v} \left(\frac{D}{W}\right), \tag{S15}$$

which scales with D/W, where D and W are the thickness (y direction) and width (z direction) of the magnetic insulator, respectively. In the present experiment, the $F_\parallel$ term is negligibly small due to $D/W \ll 1$

($D/W \sim 10^{-3}$ for the YIG substrate). By contrast, the transverse term is calculated as [S1]

$$F_{\perp} \sim -\chi_{\perp}\Phi_0^2\left(\frac{\rho_{\mathrm{v}}^2}{\xi^3}\right) = -(\Phi_0 H_{\mathrm{v}}\lambda)\chi_{\perp}\kappa^3\left(\frac{\rho_{\mathrm{v}}}{\lambda}\right)^2, \tag{S16}$$

where ξ is the coherence length and $\kappa = \lambda/\xi$. This term does not depend on the size of the magnetic insulator, and gives the dominant contribution to the vortex free energy F_{mag} when $D/W \ll 1$.

2.2 Vortex nucleation at surfaces

In the vortex-liquid phase in a type-II superconductor, vortex strings can move freely in the superconductor under a driving force. The vortex flow results in an electric field in the direction perpendicular to the vortex motion and magnetic field directions [S3]. For simplicity, we assume that the vortex flow is governed by the nucleation process of vortex strings at the surfaces of the superconductor [S1]. The nucleation rate P of a vortex is determined by the temperature T and the nucleation energy barrier ΔF [S4,S5], and given by $P = \alpha e^{-\Delta F/k_{\mathrm{B}}T}$, where α is the prefactor and k_{B} is the Boltzmann constant.

In the following, we focus on the MoGe/YIG system. The energy barriers for the vortex nucleation at the MoGe/vacuum and MoGe/YIG interfaces, ΔF_{vac} and ΔF_{YIG} respectively, are given by

$$\Delta F_{\mathrm{vac}} = \max_{\rho_{\mathrm{v}}/\lambda} G\,(\rho_{\mathrm{v}}/\lambda), \tag{S17}$$

$$\Delta F_{\mathrm{YIG}} = \max_{\rho_{\mathrm{v}}/\lambda}[G(\rho_{\mathrm{v}}/\lambda) + F_{\perp}(\rho_{\mathrm{v}}/\lambda)]\,. \tag{S18}$$

By substituting Eqs. (S6) and (S16) into Eqs. (S17) and (S18), the external magnetic field dependence of the energy barrier at each surface was calculated, as shown in Supplementary Fig. 4. The result indicates that the energy barriers at the MoGe/vacuum and MoGe/YIG interfaces are different from each other due to the interaction between the vortex and the magnetization. The difference of the energy barriers results in the difference in the vortex nucleation rate between the top and bottom surfaces of the MoGe.

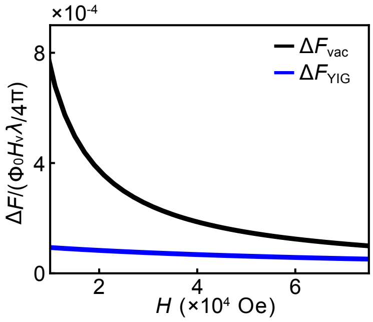

Supplementary Fig. 4 Magnetic field dependence of energy barrier at interfaces. Energy barriers ΔF_{vac} at the MoGe/vacuum interface (black solid curve) and ΔF_{YIG} at the MoGe/YIG interface (blue solid curve) as a function of the external magnetic field H. We used $\lambda = 1.04 \times 10^{-6}$ m at 4 K and $\kappa = 83$, whose values were estimated [S6] from the experimental data. The value of $4\pi\chi_{\perp}$ was set to 0.01.

2.3 Nonlinear vortex Nernst effect

We are now in a position to describe the nonlinear vortex Nernst effect in MoGe/YIG under the temperature gradient $-\nabla T$. In the presence of $-\nabla T$ in a vortex liquid phase, a vortex string feels a thermal force [S7]

$$\mathbf{f}_{\mathrm{th}} = -S_{\phi}\nabla T, \quad \text{(S19)}$$

where S_{ϕ} is the entropy carried by a vortex. This thermal force drives a vortex string with the speed $\mathbf{v}$, by which the vortex string feels a frictional force

$$\mathbf{f}_{\mathrm{f}} = -\eta\mathbf{v}, \quad \text{(S20)}$$

where η is the viscosity. In a steady state, the thermal force is in balance with the frictional force, $\mathbf{f}_{\mathrm{th}} + \mathbf{f}_{\mathrm{f}} = 0$, which leads to the terminal velocity of the vortex

$$\mathbf{v}_{\mathrm{v}} = \frac{S_{\phi}}{\eta}(-\nabla T). \quad \text{(S21)}$$

For simplicity, we assume a uniform temperature gradient in the MoGe along the thickness direction. When the temperature at the MoGe surface in contact with vacuum (YIG) is $T + \Delta T/2$ ($T - \Delta T/2$) (see Supplementary Fig. 5a), $-\nabla T$ is described as $-\nabla T = \Delta T/d(-\mathbf{e}_y)$, where T, ΔT, d, and $\mathbf{e}_y$ are the average temperature of the MoGe, the temperature difference between the top and bottom surfaces of the MoGe, the thickness of the MoGe, and a unit vector along the y axis, respectively. Due to the application of $-\nabla T$, the vortex flow $\mathbf{J}_{\mathrm{v}}(\Delta T) \propto \mathbf{v}_{\mathrm{v}}$ appears in the $-y$ direction. By contrast, when the temperature at the

MoGe/vacuum (MoGe/YIG) interface is $T - \Delta T/2$ ($T + \Delta T/2$), where $-\nabla T = -\Delta T/d(-\mathbf{e}_y)$ (see Supplementary Fig. 5b), the vortex flow $\mathbf{J}_\mathrm{v}(-\Delta T)$ in the $+y$ direction becomes finite. By using the vortex nucleation rate, we define $\mathbf{J}_\mathrm{v}(\Delta T)$ as

$$\mathbf{J}_\mathrm{v}(\Delta T) = \left[P_\mathrm{vac}\left(T + \frac{\Delta T}{2}\right) + P_\mathrm{YIG}\left(T - \frac{\Delta T}{2}\right)\right]\frac{\tau S_\phi}{\eta d}\frac{\Delta T}{d}(-\mathbf{e}_y) \tag{S22}$$

$$= J_\mathrm{v}(\Delta T)(-\mathbf{e}_y), \tag{S23}$$

where $P_\mathrm{vac}(T) = \alpha e^{-\Delta F_\mathrm{vac}/k_\mathrm{B}T}$ $[P_\mathrm{YIG}(T) = \alpha e^{-\Delta F_\mathrm{YIG}/k_\mathrm{B}T}]$ is the vortex nucleation rate at the MoGe/vacuum [MoGe/YIG] interface and τ is the time constant for the vortex number change. The vortex flow generates an electric field according to $\mathbf{E} = (\Phi_0/l)\mathbf{e}_z \times \mathbf{J}_\mathrm{v} \propto \mathbf{B} \times \mathbf{J}_\mathrm{v}$, giving rise to the vortex Nernst effect proportional to $(\Delta T)^2$. Here, l and $\mathbf{e}_z$ are the length of the MoGe film and a unit vector along the z axis, respectively. We define the electric field due to the nonlinear vortex Nernst effect as

$$E_\mathrm{N2} = \frac{(\Delta T)^2}{2}\frac{\Phi_0}{l}\frac{\partial^2}{\partial \Delta T^2} J_\mathrm{v}(\Delta T)\bigg|_{\Delta T \to 0}. \tag{S24}$$

By substituting the explicit form of $J_\mathrm{v}(\Delta T)$ into Eq. (S24), the nonlinear electric field $E_\mathrm{N2} \propto (\Delta T)^2$ is expressed as

$$E_\mathrm{N2} = -\frac{\Phi_0 S_\phi \alpha\tau}{2\eta l T}\left(\frac{\Delta F_\mathrm{YIG}}{k_\mathrm{B}T}e^{-\frac{\Delta F_\mathrm{YIG}}{k_\mathrm{B}T}} - \frac{\Delta F_\mathrm{vac}}{k_\mathrm{B}T}e^{-\frac{\Delta F_\mathrm{vac}}{k_\mathrm{B}T}}\right)\left(\frac{\Delta T}{d}\right)^2. \tag{S25}$$

When $\Delta F_\mathrm{YIG} = \Delta F_\mathrm{vac}$, E_N2 becomes zero, which means that the nonlinear vortex Nernst effect results from the asymmetry of the magnetic environment. ΔF_YIG and ΔF_vac are obtained in the calculation and the other B dependent terms in E_N2 can be estimated experimentally from the linear vortex Nernst signal E_N1 (Supplementary Fig. 6), which allows us to calculate the B dependence of E_N2 (Supplementary Fig. 7).

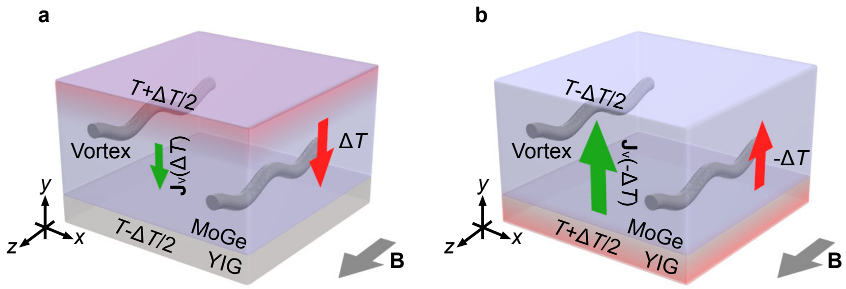

Supplementary Fig. 5 Schematic illustrations of vortex flow under temperature gradient. a When the temperature at the MoGe/vacuum interface ($T + \Delta T/2$) is higher than that at the MoGe/YIG interface ($T - \Delta T/2$), a vortex string flows in the $-y$ direction. **b** When the temperature at the MoGe/vacuum interface ($T - \Delta T/2$) is lower than that at the MoGe/YIG interface ($T + \Delta T/2$), a vortex string flows in the $+y$ direction. $\mathbf{J}_\mathrm{v}(\Delta T)$ denotes the vortex flow due to the application of the temperature gradient.

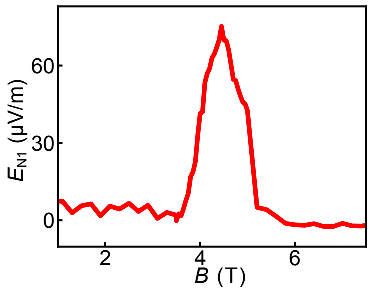

Supplementary Fig. 6 Magnetic field *B* dependence of electric field E_{N1} due to linear vortex Nernst effect. The data was experimentally obtained for the MoGe/YIG sample at $T = 4$ K. Here, the Nernst signal ($\propto B$) in the normal-conducting state ($B > 5.3$ T) is subtracted.

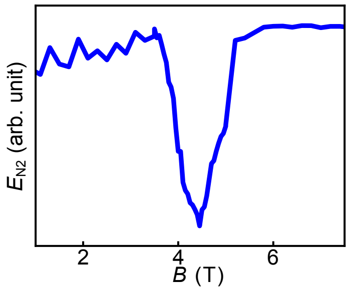

Supplementary Fig. 7 Magnetic field *B* dependence of calculated electric field E_{N2} due to nonlinear vortex Nernst effect.

Supplementary Note 3 | Comparison of thermoelectric response between MoGe/YIG and MoGe/SiO_2 samples.

We performed control experiments by comparing thermoelectric voltages for MoGe/SiO_2 (without YIG) and MoGe/YIG samples (see Supplementary Fig. 8a). Due to the small magnetic susceptibility in SiO_2, the electromagnetic asymmetry between the top and bottom surfaces of the MoGe on the SiO_2 is so small compared to the MoGe/YIG, and the nonreciprocal vortex transport is suppressed in the MoGe/SiO_2 [S1]. Supplementary Fig. 8b shows the 2ω lock-in voltage $V_{2\omega}$ as a function of the phase ϕ of the temperature difference ΔT for the MoGe/SiO_2 at each a.c. heater current $I_{\rm H}$, where the background voltage ($\propto \cos 2\phi$ and $\propto \cos 3\phi$) that is irrelevant to the nonreciprocal thermoelectric response is subtracted. In the MoGe on the SiO_2, no signal appears for all values of $I_{\rm H}$ (grey dots in Supplementary Fig. 8b), while the $V_{2\omega}$ signal for the MoGe/YIG (blue dots in Supplementary Fig. 8c) is enhanced with the increase in $I_{\rm H}$ and shows the clear $\cos\phi \sin^2(\phi/2)$ dependence with the peak amplitude $A_{2\omega}$. By plotting $A_{2\omega}$ as a function of the amplitude $A_{1\omega}(\propto \Delta T)$ of the 1ω lock-in voltage measured simultaneously, we confirmed that the value of $A_{2\omega}$ is much smaller for all values of $A_{1\omega}$ in the MoGe/SiO_2 (grey dots in Supplementary Fig. 8d) than the value of $A_{2\omega}$ $\propto A_{1\omega}^2$ in the MoGe/YIG (green dots in Supplementary Fig. 8d), excluding the sample heating from possible origins of the $V_{2\omega}$ signal $\propto \cos\phi \sin^2(\phi/2)$ in the MoGe/YIG.

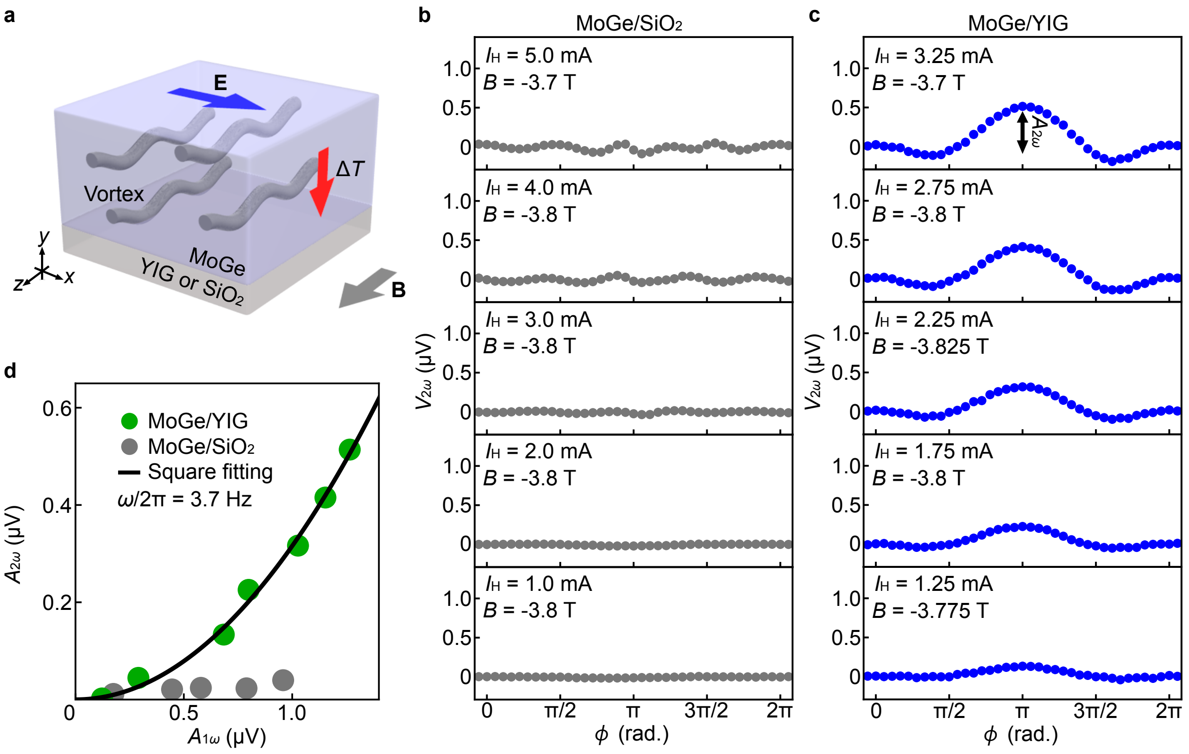

Supplementary Fig. 8 Comparison between MoGe/YIG and MoGe/SiO₂ samples. **a** A schematic illustration of the MoGe/(YIG or SiO_2) bilayer system used in the measurement. **b** The ϕ dependence of $V_{2\omega}$ measured for the MoGe/SiO_2 at each value of the a.c. heater-current amplitude $I_\mathrm{H} = (I_\mathrm{H1} + I_\mathrm{H2})$, where the currents $I_\mathrm{H1}\sin(\omega t + \phi) + I_\mathrm{DC1}$ and $I_\mathrm{H2}\sin\omega t + I_\mathrm{DC2}$ were applied to the Heater-1 and the Heater-2, respectively. **c** The ϕ dependence of $V_{2\omega}$ measured for the MoGe/YIG at each value of I_H. $A_{2\omega}$ is the peak amplitude of the $V_{2\omega}$ signal at $\phi = \pi$. **d** $A_{2\omega}$ as a function of the 1ω lock-in voltage amplitude $A_{1\omega}$ for the MoGe/YIG (green dots) and the MoGe/SiO_2 (grey dots). The black solid curve is a square fitting. The value of $\omega/2\pi$ was set to 3.7 Hz in the measurement.

Supplementary Note 4 | Evaluation of uniform heating in MoGe/YIG sample.

We estimated the influence of the sample heating by measuring the time-averaged temperature of the MoGe film. When the a.c. and d.c. currents $I_{\mathrm{H1}}\sin(\omega t+\phi)+I_{\mathrm{DC1}}$ ($I_{\mathrm{H2}}\sin\omega t+I_{\mathrm{DC2}}$) are applied to the Heater-1 (Heater-2), the temperature change ΔT_{heat} due to the heating in the MoGe film is described as

$$\Delta T_{\mathrm{heat}} = R'_1[I_{\mathrm{H1}}\sin(\omega t+\phi)+I_{\mathrm{DC1}}]^2 + R'_2(I_{\mathrm{H2}}\sin\omega t+I_{\mathrm{DC2}})^2$$

$$= T_{\mathrm{DC1}}+T_{\mathrm{DC2}}+\frac{T_{\mathrm{H1}}}{2}+\frac{T_{\mathrm{H2}}}{2}+2\sqrt{T_{\mathrm{DC1}}T_{\mathrm{H1}}}\sin(\omega t+\phi)+2\sqrt{T_{\mathrm{DC2}}T_{\mathrm{H2}}}\sin\omega t$$

$$-\frac{T_{\mathrm{H1}}}{2}\cos 2(\omega t+\phi)-\frac{T_{\mathrm{H2}}}{2}\cos 2\omega t, \quad \text{(S26)}$$

where R'_1 (R'_2) denotes a prefactor relating the Joule heating of the Heater-1 (Heater-2) to ΔT_{heat}. $T_{\mathrm{H1,DC1}}$ ($T_{\mathrm{H2,DC2}}$) is defined as $T_{\mathrm{H1,DC1}} = R'_1 I^2_{\mathrm{H1,DC1}}$ ($T_{\mathrm{H2,DC2}} = R'_2 I^2_{\mathrm{H2,DC2}}$). As shown in Eq. (S26), the amplitude of the a.c. components of ΔT_{heat} is parametrized by T_{DC1}, T_{DC2} T_{H1}, and T_{H2}. The same parameters also appear in the d.c. components of ΔT_{heat}, and their values can be estimated by measuring the time-averaged temperature of the MoGe film, $\overline{\Delta T_{\mathrm{heat}}} = T_{\mathrm{DC1}}+T_{\mathrm{DC2}}+T_{\mathrm{H1}}/2+T_{\mathrm{H2}}/2$, enabling us to check the influence of the sample heating. We evaluated the sample heating in the MoGe film on the YIG at the values of the heater currents $(I_{\mathrm{DC1}}, I_{\mathrm{DC2}}, I_{\mathrm{H1}}, I_{\mathrm{H2}}) = (3.00\ \mathrm{mA}, 2.20\ \mathrm{mA}, 1.00\ \mathrm{mA}, 0.75\ \mathrm{mA})$, where the $V_{2\omega}$ signal $\propto \cos\phi\sin^2(\phi/2)$ appears (see Supplementary Fig. 9a).

To measure the temperature T of the MoGe film, we utilized the upper critical field B_{c2} as a thermometer, since B_{c2} is very sensitive to the system temperature. As shown in Supplementary Fig. 9b, we obtained a $T(B_{\mathrm{c2}})$ curve for the MoGe (blue solid curve) from the resistance measurement. Supplementary Fig. 9c shows the magnetic field B dependence of the normalized resistivity ρ/ρ_{N} for the MoGe film when the d.c. current is applied to the Heater-1 [$(I_{\mathrm{DC1}}, I_{\mathrm{DC2}}, I_{\mathrm{H1}}, I_{\mathrm{H2}}) = (3.00\ \mathrm{mA}, 0, 0, 0)$] (black solid curve) at 4 K. B_{c2} is determined as $\sim B_{\mathrm{c2}}^{\mathrm{dc1}} = 4.65$ T from the condition $\rho/\rho_{\mathrm{N}}(B_{\mathrm{c2}}) = 0.95$ [S8], from which we estimated the temperature increase $\overline{\Delta T_{\mathrm{heat}}} = T_{\mathrm{DC1}}$ due to the d.c. current I_{DC1} application to the Heater-1 to be $T(B_{\mathrm{c2}}^{\mathrm{dc1}}) - 4\ \mathrm{K} \sim 0.26$ K. When the d.c. and a.c. currents are applied to the Heater-1 [$(I_{\mathrm{DC1}}, I_{\mathrm{DC2}}, I_{\mathrm{H1}}, I_{\mathrm{H2}}) = (3.00\ \mathrm{mA}, 0, 1.00\ \mathrm{mA}, 0)$], the value of B_{c2} decreases to $B_{\mathrm{c2}}^{\mathrm{dc1+ac1}} \sim 4.60$ T (red solid curve). From the values of B_{c2}, we estimated the temperature increase $\overline{\Delta T_{\mathrm{heat}}} = T_{\mathrm{H1}}/2$ due to the a.c. current I_{H1} application to the Heater-1 as $T(B_{\mathrm{c2}}^{\mathrm{dc1+ac1}}) - T(B_{\mathrm{c2}}^{\mathrm{dc1}}) = 0.02$ K. By performing similar measurement by using the Heater-2 (see Supplementary Fig. 9d), we obtained the complete set of $(T_{\mathrm{DC1}}, T_{\mathrm{DC2}}, T_{\mathrm{H1}}/2, T_{\mathrm{H2}}/2) =$

(0.26 K, 0.28 K, 0.02 K, 0.02 K), and estimated the amplitude of the 1ω- and 2ω-oscillating uniform sample heating as ~ 0.01 K and ~ 0.04 K at $\phi = \pi$, respectively, which is too small to explain the observed $V_{2\omega}$ signal. We also concluded that the sample heating due to the magnetocaloric effects in GGG [S9] is negligibly small.

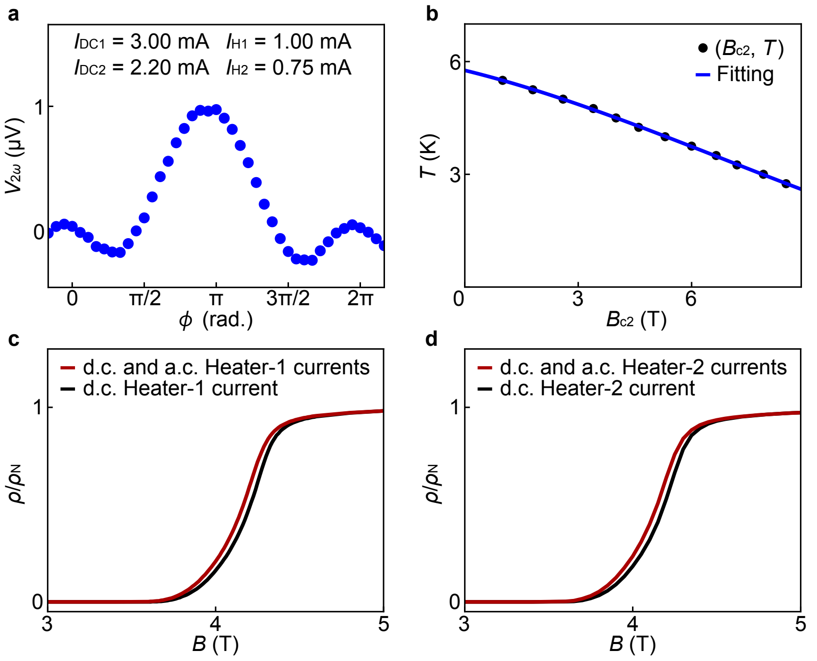

Supplementary Fig. 9 Evaluation of uniform heating in MoGe/YIG sample. a The ϕ dependence of $V_{2\omega}$ measured for the MoGe/YIG at $(I_{DC1}, I_{DC2}, I_{H1}, I_{H2}) = (3.00\text{ mA}, 2.20\text{ mA}, 1.00\text{ mA}, 0.75\text{ mA})$, where the currents $I_{H1}\sin(\omega t + \phi) + I_{DC1}$ and $I_{H2}\sin\omega t + I_{DC2}$ were applied to the Heater-1 and the Heater-2, respectively. **b** The relation between T and the upper critical field B_{c2} (black dots) of the MoGe film on the YIG. The blue solid curve is a fitting. **c** The B dependence of the normalized resistivity ρ/ρ_N for the MoGe film at $(I_{DC1}, I_{DC2}, I_{H1}, I_{H2}) = (3.00\text{ mA}, 0, 0, 0)$ (black solid curve) and $(I_{DC1}, I_{DC2}, I_{H1}, I_{H2}) = (3.00\text{ mA}, 0, 1.00\text{ mA}, 0)$ (red solid curve). Here, ρ_N is the resistivity of the film in the normal conducting state at $B = 9$ T. **d** The B dependence of ρ/ρ_N for the MoGe film at $(I_{DC1}, I_{DC2}, I_{H1}, I_{H2}) = (0, 2.20\text{ mA}, 0, 0)$ (black solid curve) and $(I_{DC1}, I_{DC2}, I_{H1}, I_{H2}) = (0, 2.20\text{ mA}, 0, 0.75\text{ mA})$ (red solid curve). The ambient temperature was set to 4 K.

Supplementary Note 5 | Influence of uniform heating in permalloy sample.

To check the influence of the sample heating, we measured the Seebeck effect in a permalloy (Py) film on SiO_2. As shown in Supplementary Fig. 10a, we applied the in-plane 1ω-oscillating temperature difference ΔT along the x axis by using the present method and measured the 1ω (2ω) lock-in voltage $V_{1\omega}$ ($V_{2\omega}$) in the same direction. Supplementary Figs. 10c and 10d show the ϕ dependence of $V_{1\omega}$ (dark blue dots) at $T = 150$ K and $T = 300$ K, respectively. $V_{1\omega}$ is proportional to $\sim$ (cos$\phi - 1$) with the amplitude $A_{1\omega}$, while no $V_{2\omega}$ signal appears (grey dots in Supplementary Figs. 10c and 10d). By measuring the T dependence of the amplitude $A_{2\omega}$ of $V_{2\omega}$, we confirmed that $A_{2\omega} \sim 0$ for all values of T (100 K $\leq T \leq$ 300 K) (grey dots in Supplementary Fig. 10b), which is not related to the temperature dependence of the Seebeck coefficient in a permalloy film [S10] (see also the T dependence of $A_{1\omega}$ in Supplementary Fig. 10b).

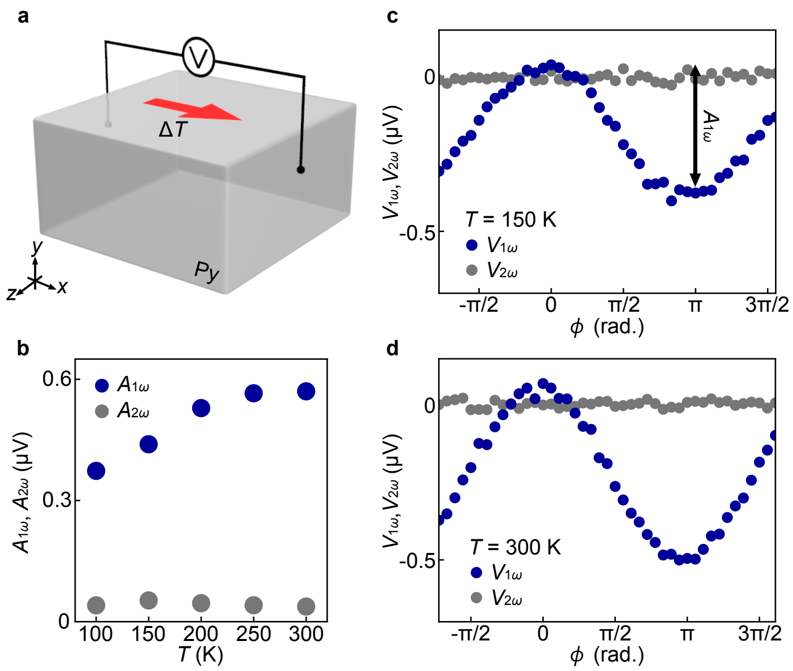

Supplementary Fig. 10 Measurement of Seebeck effect in permalloy. a A schematic illustration of the permalloy (Py) film on SiO_2 used in the measurement. **b** The T dependence of $A_{1\omega}$ (dark blue dots) and $A_{2\omega}$ (grey dots). **c** The ϕ dependence of $V_{1\omega}$ (dark blue dots) and $V_{2\omega}$ (grey dots) at $T = 150$ K. **d** The ϕ dependence of $V_{1\omega}$ (dark blue dots) and $V_{2\omega}$ (grey dots) at $T = 300$ K. $A_{1\omega}$ and $A_{2\omega}$ are defined as the difference between the maximum and minimum values of $V_{1\omega}$ and $V_{2\omega}$, respectively. The value of $\omega/2\pi$ was set to 3.7 Hz in the measurement.

Supplementary Note 6 | Evaluation of temperature inhomogeneity in MoGe/YIG sample.

We measured the spatial temperature inhomogeneity in the MoGe/YIG sample by using RuO_2-based film-thermometers [S11] and a lock-in method. As shown in Supplementary Fig. 11a, we attached the film-thermometers (4 mm × 0.5 mm × 100 nm) near (~ 1 mm) the right and left edges of the MoGe film. Supplementary Fig. 11b shows the typical T dependence of the resistivity $\rho_{\mathrm{RuO_2}}$ for the RuO_2-based film, whose value drastically increases with the decrease in T ($\lesssim$ 5 K). The resistance measurement of the film enables the sensitive detection of local temperatures. As shown in Supplementary Fig. 11a, we applied the a.c. and d.c. currents $I_{\mathrm{H1}}\sin\omega t + I_{\mathrm{DC1}}$ to the Heater-1, and measured the 1ω-oscillating (2ω-oscillating) resistance component in the RuO_2-based films by using a lock-in technique, from which we obtained the 1ω-oscillating (2ω-oscillating) temperature $T_{1\omega}^{\mathrm{right}}\sin[\omega t + \theta_{1\omega}^{\mathrm{right}}]$ and $T_{1\omega}^{\mathrm{left}}\sin[\omega t + \theta_{1\omega}^{\mathrm{left}}]$ ($T_{2\omega}^{\mathrm{right}}\sin[2\omega t + \theta_{2\omega}^{\mathrm{right}}]$ and $T_{2\omega}^{\mathrm{left}}\sin[2\omega t + \theta_{2\omega}^{\mathrm{left}}]$) at the position of the right and left RuO_2-based films, respectively. Supplementary Fig. 11c shows the $I_{\mathrm{H1}}I_{\mathrm{DC1}}$ dependence of $T_{1\omega}^{\mathrm{right}}$ (red triangles) and $T_{1\omega}^{\mathrm{left}}$ (pink rectangles), whose values are proportional to $I_{\mathrm{H1}}I_{\mathrm{DC1}}$. By comparing $T_{1\omega}^{\mathrm{right}}$ and $T_{1\omega}^{\mathrm{left}}$, we confirmed that $T_{1\omega}^{\mathrm{right}}$ and $T_{1\omega}^{\mathrm{left}}$ are almost the same in the entire range of $I_{\mathrm{H1}}I_{\mathrm{DC1}}$. As shown in Supplementary Fig. 11c, the phase of the oscillation is also the same, $\theta_{1\omega}^{\mathrm{right}} = \theta_{1\omega}^{\mathrm{left}}$, between the local temperatures near the right and left edges of the MoGe film (black triangles and grey rectangles). We also confirmed that $T_{2\omega}^{\mathrm{right}} = T_{2\omega}^{\mathrm{left}}$ and $\theta_{2\omega}^{\mathrm{right}} = \theta_{2\omega}^{\mathrm{left}}$ (see Supplementary Fig. 11d), and the same condition ($T_{1\omega/2\omega}^{\mathrm{right}} = T_{1\omega/2\omega}^{\mathrm{left}}$ and $\theta_{1\omega/2\omega}^{\mathrm{right}} = \theta_{1\omega/2\omega}^{\mathrm{left}}$) is satisfied when we used the Heater-2 (see Supplementary Figs. 11e and 11f), indicating that the temperature inhomogeneity is negligibly small along the sample length in the present method.

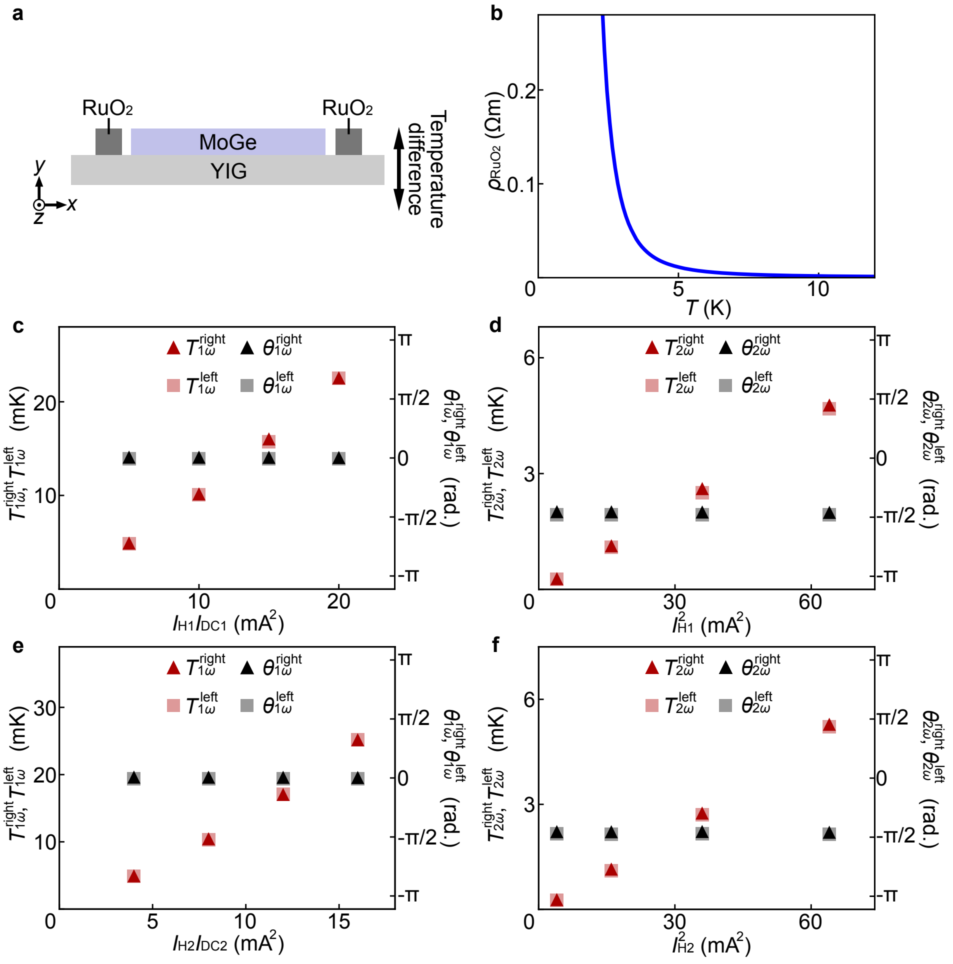

Supplementary Fig. 11 Measurement of local temperature in MoGe/YIG sample. a A schematic illustration of the measurement. Temperature difference was applied to the MoGe/YIG along the thickness direction by applying the a.c. and d.c. currents $I_{\mathrm{H1}}\sin\omega t + I_{\mathrm{DC1}}$ to the Heater-1 or applying the currents $I_{\mathrm{H2}}\sin\omega t + I_{\mathrm{DC2}}$ to the Heater-2. The 1ω-oscillating (2ω-oscillating) temperature components $T_{1\omega}^{\mathrm{right}}\sin[\omega t + \theta_{1\omega}^{\mathrm{right}}]$ and $T_{1\omega}^{\mathrm{left}}\sin[\omega t + \theta_{1\omega}^{\mathrm{left}}]$ ($T_{2\omega}^{\mathrm{right}}\sin[2\omega t + \theta_{2\omega}^{\mathrm{right}}]$ and $T_{2\omega}^{\mathrm{left}}\sin[2\omega t + \theta_{2\omega}^{\mathrm{left}}]$) were measured by using the right and left RuO_2-based film-thermometers, respectively. **b** The T dependence of the resistivity $\rho_{\mathrm{RuO_2}}$ for the RuO_2-based film. **c** The $I_{\mathrm{H1}}I_{\mathrm{DC1}}$ dependence of $T_{1\omega}^{\mathrm{right}}$ (red triangles), $T_{1\omega}^{\mathrm{left}}$ (pink rectangles), $\theta_{1\omega}^{\mathrm{right}}$ (black triangles), and $\theta_{1\omega}^{\mathrm{left}}$ (grey rectangles). **d** The I_{H1}^2 dependence of $T_{2\omega}^{\mathrm{right}}$ (red triangles), $T_{2\omega}^{\mathrm{left}}$ (pink rectangles), $\theta_{2\omega}^{\mathrm{right}}$ (black triangles), and $\theta_{2\omega}^{\mathrm{left}}$ (grey rectangles). **e** The $I_{\mathrm{H2}}I_{\mathrm{DC2}}$ dependence of $T_{1\omega}^{\mathrm{right}}$ (red triangles), $T_{1\omega}^{\mathrm{left}}$ (pink rectangles), $\theta_{1\omega}^{\mathrm{right}}$ (black triangles), and $\theta_{1\omega}^{\mathrm{left}}$ (grey rectangles). **f** The I_{H2}^2 dependence of $T_{2\omega}^{\mathrm{right}}$ (red triangles), $T_{2\omega}^{\mathrm{left}}$ (pink rectangles), $\theta_{2\omega}^{\mathrm{right}}$ (black triangles), and $\theta_{2\omega}^{\mathrm{left}}$ (grey rectangles). The value of $\omega/2\pi$ was set to 3.7 Hz in the measurement.

Supplementary Note 7 | Out-of-plane and in-plane temperature gradients in MoGe/YIG sample.

We measured the temperature difference generated by the present method in the MoGe/YIG sample. As shown in Supplementary Fig. 12a, we applied a.c. and d.c. currents to the two heaters, and measured the 1ω- and 2ω-oscillating components ($\Delta T_{1\omega}^{\mathrm{ac}}$ and $\Delta T_{2\omega}^{\mathrm{ac}}$, respectively) of the generated temperature difference with three RuO_2-based film-thermometers, each of which is located at the top-right, top-left, and bottom-right surfaces of the sample. Supplementary Figs. 12b and 12c show the ϕ dependence of the temperature difference amplitude $\Delta T_{1\omega}$ of $\Delta T_{1\omega}^{\mathrm{ac}}$ and the amplitude $\Delta T_{2\omega}$ of $\Delta T_{2\omega}^{\mathrm{ac}}$ along the sample thickness, respectively (black dots). Here, $\Delta T_{1\omega}$ and $\Delta T_{2\omega}$ are normalized by the thickness s of the sample. When $\phi = 0$, $\Delta T_{1\omega}$ and $\Delta T_{2\omega}$ take the minimum values due to the cancellation of the 1ω- and 2ω-oscillating temperature differences generated from the two heaters, respectively. At $\phi = \pi$, the value of $\Delta T_{1\omega}$ is maximized while $\Delta T_{2\omega}$ is kept almost zero, which demonstrates that the 1ω-oscillating (2ω-oscillating) temperature difference is enhanced (remains cancelled out) between the heaters. We also confirmed that the observed ϕ dependence of $\Delta T_{1\omega}$ and $\Delta T_{2\omega}$ agrees with the formula expected from $\Delta T_{1\omega}^{\mathrm{ac}} \propto [\sin(\omega t + \phi) - \sin \omega t]$ and $\Delta T_{2\omega}^{\mathrm{ac}} \propto [\cos 2(\omega t + \phi) - \cos 2\omega t]$, respectively (red curves). The results demonstrate again that the amplitude of the temperature difference is controlled by tuning ϕ in the present method. As shown in Supplementary Figs. 12b and 12c, we also measured the 1ω- and 2ω-oscillating temperature gradients $\Delta T_{1\omega}^{x}/u$ and $\Delta T_{2\omega}^{x}/u$ along the sample length (grey dots), whose values are much smaller than those of $\Delta T_{1\omega}/s$ and $\Delta T_{2\omega}/s$, respectively (black dots). Furthermore, the Seebeck coefficient S ($\lesssim 0.05$ μV/K) is much smaller than the Nernst coefficient (~ 10 μV/K) in MoGe films [S12], and the T dependence of S is also small. We confirmed this characteristic for the present sample (MoGe films fabricated on YIG). From the values of $\Delta T_{1\omega}^{x}/u$ and the amplitude $(\Delta T_{\mathrm{heat}})_{1\omega}$ of the 1ω-oscillating component of ΔT_{heat}, we estimated the contamination of the T dependence of S in $V_{2\omega}$: $\partial S/\partial T \times (\Delta T_{\mathrm{heat}})_{1\omega} \times \Delta T_{1\omega}^{x}/u \times l \lesssim 10^{-5}$ μV, which is too small to explain the observed $V_{2\omega}$ signal for the MoGe/YIG. Here l is the length of the MoGe film.

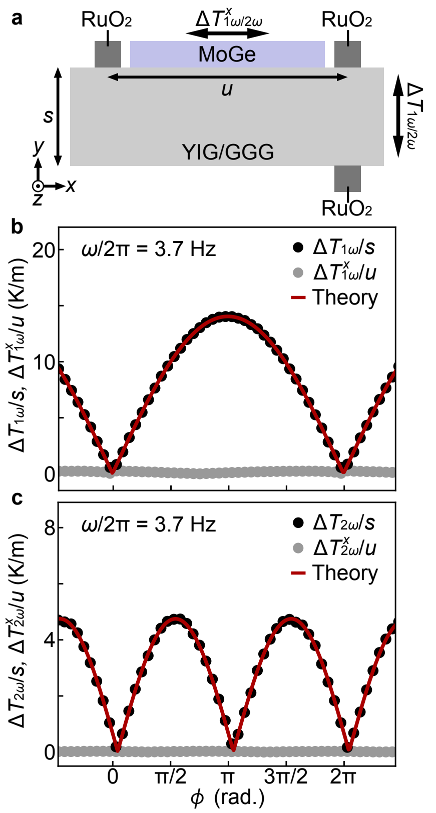

Supplementary Fig. 12 Measurement of out-of-plane and in-plane temperature gradients in MoGe/YIG sample. a A schematic illustration of the measurement. The 1ω- and 2ω-oscillating temperature differences with the amplitude $\Delta T_{1\omega}$ and $\Delta T_{2\omega}$, respectively, were applied to the MoGe/YIG along the thickness direction (y direction) by applying the a.c. and d.c. currents $I_{\mathrm{H1}}\sin(\omega t+\phi)+I_{\mathrm{DC1}}$ ($I_{\mathrm{H2}}\sin\omega t+I_{\mathrm{DC2}}$) to the Heater-1 (Heater-2), and measured with RuO_2-based film-thermometers. The 1ω- and 2ω-oscillating temperature differences along the sample length (x direction) with the amplitude $\Delta T_{1\omega}^{x}$ and $\Delta T_{2\omega}^{x}$, respectively, were also measured simultaneously. s is the thickness of the substrate and u is the distance between the right and left RuO_2-based films. **b** The ϕ dependence of $\Delta T_{1\omega}/s$ (black dots) and $\Delta T_{1\omega}^{x}/u$ (grey dots). **c** The ϕ dependence of $\Delta T_{2\omega}/s$ (black dots) and $\Delta T_{2\omega}^{x}/u$ (grey dots). Red solid curves are theoretical fittings. The value of $\omega/2\pi$ was set to 3.7 Hz in the measurement.

Supplementary Note 8 | Out-of-plane temperature gradient in permalloy sample.

By measuring the anomalous Nernst effect for a Py film on SiO_2 as a reference sample, we checked the validity of the present method. We applied 1ω- and 2ω-oscillating temperature differences to the film in the thickness direction and measured the 1ω and 2ω lock-in voltages $V_{1\omega}$ and $V_{2\omega}$ along the film length, respectively. The in-plane magnetic field B was applied perpendicular to the film length. Supplementary Fig. 13a shows the B dependence of $V_{1\omega}$ at $\phi = 0$ (grey dots) and $\phi = \pi$ (black dots). At $\phi = 0$, $V_{1\omega}$ is almost zero in the entire range of B. When the value of ϕ is changed from 0 to π, a clear anomalous Nernst signal appears in $V_{1\omega}$. In contrast, as shown in Supplementary Fig. 13b, the value of $V_{2\omega}$ remains unchanged (~ 0) between $\phi = 0$ (grey dots) and $\phi = \pi$ (black dots). The result supports that only the 1ω-oscillating temperature difference is enhanced by changing $\phi = 0$ to $\phi = \pi$ in the present method.

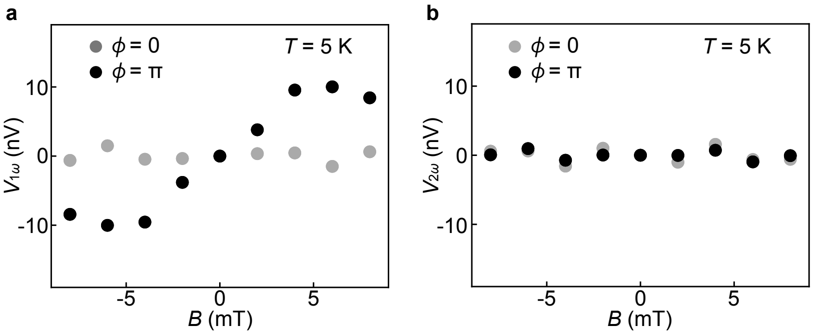

Supplementary Fig. 13 Measurement of Nernst effect in permalloy film. a, b The B dependence of (**a**) $V_{1\omega}$ and (**b**) $V_{2\omega}$ at $\phi = 0$ (grey dots) and $\phi = \pi$ (black dots). The value of T was set to 5 K.

Supplementary Note 9 | Frequency dependence of thermoelectric voltage in MoGe/YIG sample.

Supplementary Figs. 14a-14g show the ϕ dependence of $V_{2\omega}$ for the MoGe/YIG at $\omega/2\pi = 1.7$ Hz, 3.7 Hz, 5.7 Hz, 7.7 Hz, 9.7 Hz, 11.7 Hz, and 13.7 Hz, respectively. $V_{2\omega}$ exhibits the characteristic ϕ dependence $[\propto \cos\phi\sin^2(\phi/2)]$ for all values of ω, which indicates that the nonlinear thermoelectric effect $\propto (\Delta T)^2$ appears at the ω range.

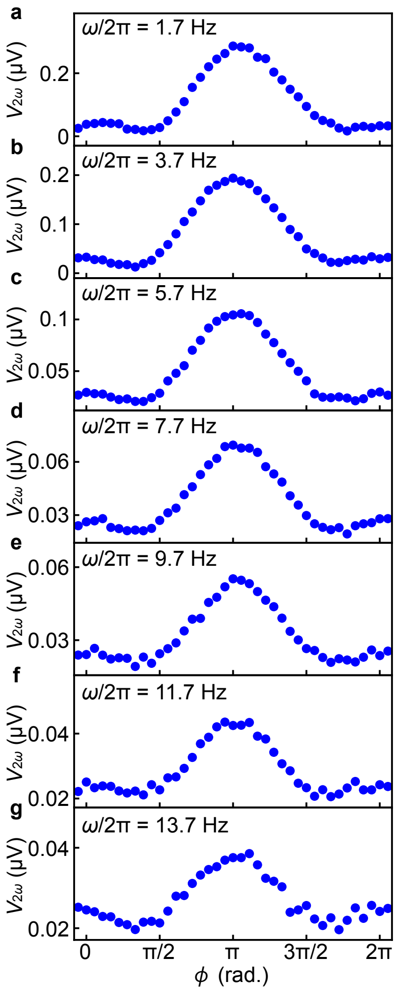

Supplementary Fig. 14 Frequency dependence of nonlinear thermoelectric voltage in MoGe/YIG. a-g The ϕ dependence of $V_{2\omega}$ at $\omega/2\pi =$ (**a**) 1.7 Hz, (**b**) 3.7 Hz, (**c**) 5.7 Hz, (**d**) 7.7 Hz, (**e**) 9.7 Hz, (**f**) 11.7 Hz, and (**g**) 13.7 Hz.

Supplementary References

S1. Lustikova, J. *et al*. Vortex rectenna powered by environmental fluctuations. *Nat. Commun*. **9**, 4922 (2018).

S2. Samokhvalov, A. V. Vortex loops entry into type-II superconductors. *Physica C* **259**, 337-348 (1996).

S3. Tinkham, M. *Introduction to Superconductivity* (Dover Publications, New York, 1996).

S4. Bean, C. P. & Livingston, J. D. Surface barrier in type-II superconductors. *Phys. Rev. Lett.* **12**, 14-16 (1964).

S5. Halperin, B. I., Refael, G. & Demler, E. Resistance in superconductors. *Int. J. Mod. Phys. B* **24**, 4039-4080 (2010).

S6. Kes, P.H. & Tsuei, C.C. Two-dimensional collective flux pinning, defects, and structural relaxation in amorphous superconducting films. *Phys. Rev. B* **28**, 5126 (1983).

S7. Huebener, R. P. *Magnetic Flux Structures in Superconductors* (Springer, Berlin, 1979).

S8. Okuma, S., Inoue, J. & Kokubo, N. Broadband noise across the mode-locking freezing in driven vortex matter. *Int. J. Mod. Phys. B* **21**, 3379-3386 (2007).

S9. Wikus P., Burghart G. & Figueroa-Feliciano, E. Optimum operating regimes of common paramagnetic refrigerants. *Cryogenics* **51**, 555-558 (2011).

S10. Hojem, A., Wesenberg, D. & Zink, B. L. Thermal spin injection and interface insensitivity in permalloy/aluminum metallic nonlocal spin valves. *Phys. Rev. B* **94**, 024426 (2016).

S11. Kikkawa, T., Kiguchi, H., Kaverzin, A. A., Takahashi, R. & Saitoh E. Cryogenic spin Peltier effect detected by a RuO_2-AlO_x on-chip microthermometer. *Phys. Rev. Applied* **20**, 054006 (2023).

S12. Wu, Y., Roy, A., Dutta, S., Jesudasan, J., Raychaudhuri, P. & Frydman A. Nernst sign reversal in the hexatic vortex phase of weakly disordered a-MoGe thin films. *Phys. Rev. Lett.* **132**, 026003 (2024).
